# Regulation of RNA N^6^-methyladenosine modification and its emerging roles in skeletal muscle development

**DOI:** 10.7150/ijbs.56251

**Published:** 2021-04-12

**Authors:** Jiju Li, Yangli Pei, Rong Zhou, Zhonglin Tang, Yalan Yang

**Affiliations:** 1 Guangdong Provincial Key Laboratory of Animal Molecular Design and Precise Breeding, Key Laboratory of Animal Molecular Design and Precise Breeding of Guangdong Higher Education Institutes, School of Life Science and Engineering, Foshan University, Foshan 528231, Guangdong, China.; 2 Shenzhen Branch, Guangdong Laboratory for Lingnan Modern Agriculture, Agricultural Genomics Institute at Shenzhen, Chinese Academy of Agricultural Sciences, Shenzhen, 518124, China.; 3 State Key Laboratory of Animal Nutrition; Key Laboratory of Animal Genetics Breeding and Reproduction, Ministry of Agriculture and Rural Affairs, Institute of Animal Science, Chinese Academy of Agricultural Sciences, Beijing 100193, China.

**Keywords:** RNA N6-methyladenosine, development, skeletal muscle, myogenesis

## Abstract

N^6^-methyladenosine (m^6^A) is one of the most widespread and highly conserved chemical modifications in cellular RNAs of eukaryotic genomes. Owing to the development of high-throughput m^6^A sequencing, the functions and mechanisms of m^6^A modification in development and diseases have been revealed. Recent studies have shown that RNA m^6^A methylation plays a critical role in skeletal muscle development, which regulates myoblast proliferation and differentiation, and muscle regeneration. Exploration of the functions of m^6^A modification and its regulators provides a deeper understanding of the regulatory mechanisms underlying skeletal muscle development. In the present review, we aim to summarize recent breakthroughs concerning the global landscape of m^6^A modification in mammals and examine the biological functions and mechanisms of enzymes regulating m^6^A RNA methylation. We describe the interplay between m^6^A and other epigenetic modifications and highlight the regulatory roles of m^6^A in development, especially that of skeletal muscle. m^6^A and its regulators are expected to be targets for the treatment of human muscle-related diseases and novel epigenetic markers for animal breeding in meat production.

## Introduction

RNA post-transcriptional modifications exist widely in eukaryotic genomes and play important roles in many biological processes such as development, reproduction, and disease 1, 2. To date, more than 100 types of chemical modifications have been identified in cellular RNAs of eukaryotic genomes, including N^7^-methylguanine (m^7^G), N^6^-methyladenosine (m^6^A), 5-methylcytosine (m^5^C), N^1^-methyl-methylguanine (m^1^A), pseudouridine (ψ), and 5'-O-methylation (Nm). Among these, the m^6^A modification is the most common and abundant form of RNA methylation and is mainly located at the nitrogen atom in the sixth position of adenosine 3. The m^6^A modification is enriched around the start and stop codons/3′-untranslated regions (UTRs) containing an RRACH consensus motif (where R is G or A, and H is A, C, or U), which is conserved across species 4. The m^6^A modification was originally discovered in bacterial DNA 5 but was found for the first time in the 1970s in mRNA of Novikoff hepatoma cells, with an average of three to five m^6^A sites per mRNA unit 6. In 2011, the first m^6^A demethylase fat mass and obesity-associated (FTO) gene was discovered, indicating for the first time that the m^6^A modification was dynamic and reversible 7. This study became a milestone in RNA epigenetics research and initiated a surge in the investigation of m^6^A methylation. Owing to the development of high-throughput sequencing technology in combination with immunoprecipitation methods, the genome-wide presence of m^6^A modification in RNAs can be profiled and its dynamic regulation is explored 3, 8-12, greatly accelerating the discovery the m^6^A modification sites in genome of various species, and making it possible to further study the function and mechanism of m^6^A in development and other biological processes.

Skeletal muscle development is a complex process involving multiple stages of proliferation and differentiation. The current research on skeletal muscle development is mainly focused on molecular regulation at the levels of protein-coding genes and non-coding RNAs (such as miRNAs, lncRNAs, and circRNAs) 13. The post-transcriptional mechanisms of skeletal muscle development remain largely unknown. Recent studies have suggested that m^6^A methylation regulates myoblast proliferation and differentiation during myogenesis and plays an important role in key biological processes and regulatory pathways during skeletal muscle development 14. In the present paper, we summarize the current studies on the global landscape of m^6^A modifications in mammals and review the biological functions and mechanisms of enzymes regulating m^6^A RNA methylation. We emphasize the regulatory roles of m^6^A modifications in development, especially that of skeletal muscle, which may help to further understand the mechanism underlying the epigenetic regulation of skeletal muscle development and muscle-related diseases at the post-transcriptional level.

## Genome-wide m^6^A methylation map in mammals

Genome-wide m^6^A methylation studies have shown that m^6^A methylation exists widely in the genomes of prokaryotes, eukaryotes, and viruses. In addition to those in mRNAs, a large number of m^6^A methylation modifications have been found in non-coding RNAs (ncRNAs), such as long non-coding RNAs (lncRNAs), circular RNAs (circRNAs), transfer RNAs (tRNAs), ribosomal RNAs (rRNAs), and splice RNAs 6, 15. It has been reported that m^6^A-modified adenosine accounts for 0.1-0.4% of the total adenosine content of RNA, with approximately 12,000 m^6^A methylation sites existing in the genome 8. In the human genome, m^6^A modifications exist on more than 7,000 mRNAs and 300 ncRNAs, and are located in the 3'UTR of mRNA and the stop codon region of coding sequences (CDS), distributed mainly within G(M6A)C (70%) or A(M6A)C (30%) conservative sequences 3. Recent studies have also suggested that the m^6^A modification is widespread in circRNAs and is expressed in a highly cell-specific pattern. Despite sharing m^6^A readers (binding proteins) and writers (methyltransferases), m^6^A circRNAs are frequently derived from exons that are not methylated in mRNAs 16. Moreover, m^6^A modifications in circRNAs have been reported to modulate circRNA biogenesis and function 17. Studies have shown that the genome-wide distribution of the m^6^A modification in embryonic stem cells and somatic cells is highly conserved across species. Approximately 70% of the transcripts modified by m^6^A in humans are also modified by m^6^A in mice, and roughly 46% of these sites are identical, indicating that m^6^A displays a certain degree of species conservation 8. The whole-transcriptome landscape of m^6^A modifications in human fetal tissues uncovered high tissue variation. It was found that the m^6^A modification is positively correlated with the dynamic balance of gene expression and preferentially occupies genes rich in CpG promoters that regulate RNA transcript m^6^A, indicating that m^6^A is widely regulated by human genetic variation and promoter activity 18. A study on the m^6^A modification map of the cerebellum in postnatal mice showed that m^6^A methylation and demethylation occur widely in both proliferating and fully differentiated neurons during differentiation *in vivo*. Functional analysis of genes containing on/off switches for m^6^A has demonstrated that m^6^A is a prerequisite for these genes to perform their functions at each developmental stage. RNA m^6^A methylation is controlled in a precise spatiotemporal manner and is involved in the regulation of postnatal cerebellar development in mice 19. Taken together, the results shed light on the prevalence and characterizations of RNA m^6^A modification in different tissues, development stages and species, and provide a rich resource for in-depth exploring the mechanistic role of m^6^A in various biological processes.

## Enzymes regulating RNA m^6^A methylation

**Figure 1:**
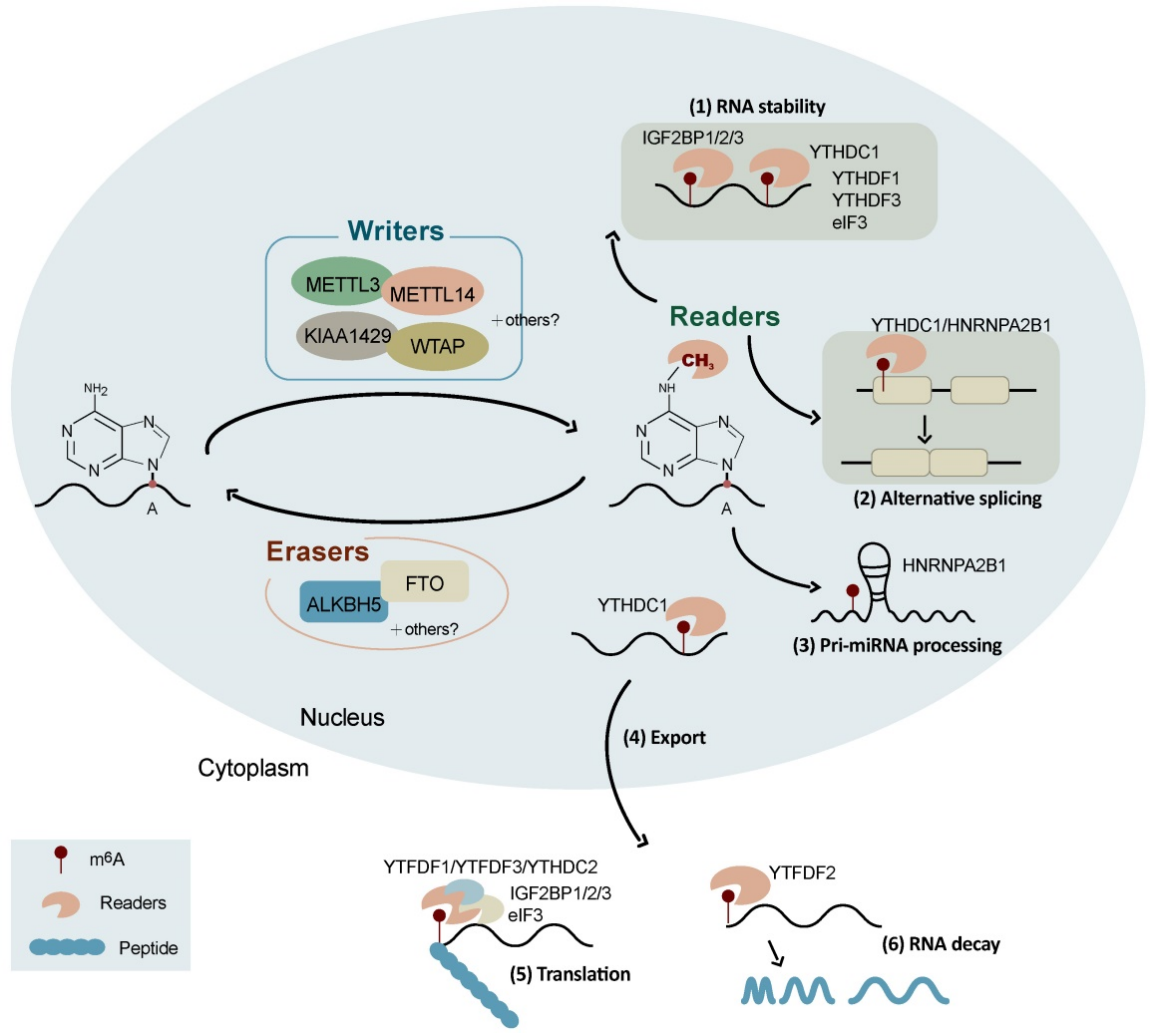
** Schematic representation of the regulation and functions of m^6^A RNA modification.** The reversible m^6^A RNA modification is dynamically regulated by m^6^A methyltransferases (writers) and demethylases (erasers), and recognized binding proteins (readers). Depending on the cellular context and their position in RNAs, m^6^A is involved in a number of RNA processes including alternative splicing, RNA stability, pri-miRNA processing, RNA nuclear export, RNA decay, and translation.

Similar to DNA methylation, the RNA m^6^A modification is dynamic and reversible. The m^6^A modification is generated by the METTL3/14 methyltransferase complex using the co-factor S-adenosyl methionine (SAM) as the major biological methyl donor 20. The m^6^A modification is finely balanced through an interplay among m^6^A methyltransferases (writers) 21, demethylases (erasers), and binding proteins (readers) (Figure 1) 7, 22.

RNA methyltransferases include methyltransferase-like 3 (METTL3), methyltransferase-like 14 (METTL14), Wilms tumor 1-associated protein (WTAP), Vir-like m^6^A methyltransferase-associated (VIRMA) protein (also named as KIAA1420), and RNA binding motif protein 15 (RBM15) 9, 20, 23-26. METTL3 and METTL14 act as a stable heterodimer core of the RNA m^6^A methyltransferase complex to recognize methylated adenosine bases on RNA and perform catalytic function 20, 27. WTAP, a mammalian splicing factor, is the third indispensable component of the m^6^A methyltransferase complex *in vivo* 28. WTAP itself has no methylation activity; however, its interaction with the METTL3/14 heterodimer can significantly affect cellular m^6^A deposition 20, 28. WTAP and METTL3/14 colocalize in nuclear speckles and participate in RNA m^6^A methylation 29. In addition, recent studies have found that the zinc finger structure (ZC3H13) is a critical regulator of RNA m^6^A methylation and helps to anchor the Zc3h13-WTAP-Virilizer-Hakai complex, an important regulatory component of RNA m^6^A, in the nucleus 30.

RNA demethylases (erasers) can reverse the methylation at N^6^A sites 7, 22, among which FTO was the first to be discovered 7. It has since been shown that inhibition of FTO can increase the level of m^6^A modification across all mRNAs 31. In most cell lines, FTO is mainly localized in the nucleus and mediates approximately 5-10% of the total mRNA m^6^A demethylation, while in some leukemia cells, FTO mediates up to 40% of the total mRNA m^6^A demethylation 32. AlkB homolog 5 (ALKHB5) was the second demethylase to be discovered, the gene for which belongs to the same *Alkb* gene family as that for FTO; however, its demethylase function was not determined until 2013, when it was shown to remove the m^6^A modification from single-stranded DNA and RNA 22. The actions of FTO and ALKHB5 indicate that m^6^A modification is a dynamic and reversible process.

The downstream functions of m^6^A are mediated by reader proteins that recognize m^6^A and regulate mRNA processing 33, which mainly include YT521-B homology (YTH) domain-containing family 1-3 (YTHDF1-3), YTH domain-containing 1-2 (YTHDC1-2), heterogeneous ribonucleoproteins (HNRNPA2B1, HNRNPC), insulin-like growth factor 2 mRNA binding proteins (IGF2BP1/2/3), and eIF3 34. Remarkably, the m^6^A modification-related enzymes are also reported to be regulated by other genes and small-molecule compounds 35-39. These findings greatly expand our understanding of the regulation of m^6^A, which might help to develop new targets for diseases and breeding.

## Interplay between m^6^A methylation and other epigenetic modifications

Genome-wide m^6^A methylation analysis has suggested that there is an interaction between m^6^A modification and other post-transcriptional regulatory modifications 40-43. m^6^A methylation and adenosine-inosine (A-to-I) editing are two distinct and abundant RNA modifications at adenosine, between which there exists a negative correlation. A global A-to-I difference has been observed between m^6^A-positive and m^6^A-negative RNA populations. Knockdown of m^6^A writers or erasers leads to a global change in A-to-I editing 40. The landscapes and distribution patterns of the m^6^A and m^6^Am methylomes across human and mouse tissues suggest that these two RNA modifications are highly specific in the brain. The overall m^6^A and m^6^Am levels are partially correlated with those of their writers and erasers 41. Loss of YTHDF2 stabilizes lysine demethylase 6B (KDM6B), promoting histone H3 lysine-27 trimethylation (H3K27me3) of multiple proinflammatory cytokines and subsequently enhancing their transcription. Moreover, H3K27me3 is also a barrier to m^6^A modification during transcription. These results suggest an interplay between m^6^A and H3K27me3 during bacterial infection 42. In addition, a recent study suggested that the gut microbiota affects the m^6^A epitranscriptome of mouse cecum and liver, mediating pathways related to metabolism, inflammation, and antimicrobial response 43. Overall, these findings suggest a previously underappreciated interplay between m^6^A and other epigenetic modifications. Further studies are required to explore the interaction mechanisms between various epigenetic modifications in more detail with improved approaches and analysis methods.

## Functions of m^6^A methylation in RNA metabolism

Substantial progress in the knowledge of the functions of m^6^A modification has been witnessed in recent years. It is well known that m^6^A is involved in a number of RNA processes including RNA nuclear export, splicing, stability, degradation, and translation (Figure 1) 32. The initiation of mRNA export from the nucleus to the cytoplasm by m^6^A modification is an important way of regulating gene expression, which is mediated by METTL3 44, ALKBH5 22, and YTHDC1 45. The combination of YTHDC1, SRF3, and NXF1 (nuclear RNA output factor) can promote the nucleation of m^6^A-modified mRNA 46. The main role of ALKBH5 is to demethylate m^6^A-modified mRNA and exerts its function by participating in the regulation of mRNA nucleation and other metabolic processes 22.

The m^6^A modification is also a splicing regulator and significantly affects alternative splicing patterns (Figure 1). It has been reported that mRNAs undergoing alternative splicing have greater numbers of METTL3 binding and m^6^A modification sites 8. FTO targets pre-mRNAs in intronic regions and regulates alternative splicing and 3'-end processing 47. The demethylation activity of ALKBH5 affects the nuclear speckle localization of several splicing factors and alters more than 3,000 mRNA isoforms, suggesting a role in splicing 22. m^6^A modifications alter the structure of mRNAs and lncRNAs, affecting their binding to HNRNPC and therefore influencing the abundance and alternative splicing of target mRNAs in the nucleus 48. YTHDC1 affects the nucleation and splicing of m^6^A-modified mRNA mainly by binding to specific m^6^A sites or splicing regulatory factors 49. HNRNPA2B1 can directly bind to a set of nuclear transcripts within the transcriptome and regulate their alternative splicing in a manner similar to that of METTL3 50. HNRNPA2B1 also acts as a nuclear reader of the m^6^A marker and partially mediates its effect on primary microRNA processing and alternative splicing 50. Moreover, m^6^A has been reported to be associated with alternative polyadenylation (APA) usage, which is coupled to the splicing of the last intron 51.

Recent studies have suggested that the m^6^A modification promotes mRNA translation through several distinct mechanisms (Figure 1) 52 including the YTHDF1-eIF3 pathway 52, cap-independent translation 53, 54, and IGF2BP-mediated translation 34. YTHDF1 can bind to m^6^A-modified mRNA and recruit translation initiation factor complex eIF3 to promote mRNA translation, which is mediated by METTLE3 methylase activity and the YTHDF1-eIF3 pathway 55, 56. YTHDF1 also recognizes m^6^A-modified lysosomal protease mRNA, the binding of which promotes the translation of lysosomal protease, thus inhibiting its antigenic cross-presentation ability 57. YTHDF2 can affect RNA stability and accelerate the decay of m^6^A-modified RNAs 58, 59. It has been found that the YTH and R3H domains of YTHDC2 facilitate its binding to cellular RNA and interact with small ribosomes, promoting mRNA translation 60. YTHDF3 facilitates translation and promotes protein synthesis in synergy with YTHDF1, and affects methylated mRNA decay mediated by YTHDF2 61. The m^6^A-driven translation of circRNAs requires initiation factor eIF4G2 (a non-canonical eIF4G protein) and YTHDF3, which is enhanced by METTL3/14 and inhibited by FTO 62. IGF2BPs enhance mRNA stability and translation in an m^6^A-dependent manner. In contrast to the mRNA decay-promoting function of YTHDF2, IGF2BPs promote the stability and storage of their target mRNAs, globally affecting gene expression 34. The m^6^A modification also promotes the initiation of miRNA biogenesis and regulates nuclear mRNA processing events 63. In addition, the m^6^A modification controls the RNA structure-dependent accessibility of RNA binding motifs to affect RNA-protein interactions for biological regulation, which is referred to as the m^6^A switch and widely exists throughout the transcriptome 48.

## RNA m^6^A modification in developmental processes

The m^6^A modification is involved in the control of delicate gene expression patterns and regulates many developmental processes. During stem cell development, m^6^A can accurately regulate stem cell differentiation and reprogramming by modulating the expression of genes involved in the corresponding processes 64. There exist several METTL3 binding sites on the mRNA of pluripotent factor NANOG in mouse embryonic stem cells. Knockout of METTL3 decreases the m^6^A methylation level of NANOG and suppresses the self-renewal and differentiation ability of embryonic stem cells. Moreover, METTL3 is also a regulator of murine naïve pluripotency termination. The m^6^A modification predominantly and directly reduces the mRNA stability of key naïve pluripotency-promoting transcripts 65. Conditional knockout of METTL3 in bone marrow mesenchymal stem cells (MSCs) results in pathological features such as osteoporosis in mice 66. Overexpression of METTL3 in bone marrow MSCs has a protective effect against estrogen-deficiency osteoporosis in mice. Knockout of METTL3 also reduces the translation efficiency of the MSC distributor Pth1r, inhibiting osteogenesis and adipogenesis induced by PTH *in vivo* 66. LincRNA1281 can regulate the differentiation of mouse embryonic stem cells through a competitive endogenous RNA (ceRNA) model mediated by m^6^A methylation 67. METTL3 is also reported as a negative regulator of autophagy of cardiomyocytes. METTL3 and ALKBH5 can oppositely regulate m^6^A modification of transcription factor EB (TFEB) mRNA. In turn, TFEB regulates the expression levels of METTL3 and ALKBH5 in opposite directions, which uncovers a critical link between METTL3-ALKBH5 and autophagy 68.

The m^6^A modification is highly enriched in the mammalian brain 3 and is essential for brain development and function 69. Methylated RNA immunoprecipitation sequencing (MeRIP-seq) analysis has suggested that thousands of coding genes and hundreds of non-coding genes are modified by m^6^A in brain tissue 3. The transcriptomic profiles of m^6^A are spatially regulated in different brain regions. The m^6^A modification is highly enriched in genes related to neuronal functions, such as synaptic release and uptake 70. Moreover, m^6^A signaling is required for robust axon regeneration in the adult mammalian nervous system. METTL3 knockdown results in a prolonged cell cycle in cortical neural progenitor cells and decreased differentiation of radial glial cells. Knockout of METTL14 in mouse embryos leads to prolongation of cortical neurogenesis to the postnatal stage 71. These results indicate a pivotal regulatory role of m^6^A during brain development.

The m^6^A modification has also been reported to be a critical regulator of adipogenesis. In rat adipocytes, the m^6^A modification strongly stimulates glucose oxidation 72. FTO-mediated m^6^A demethylation is closely related to upregulated adipogenesis, fat mass, and body weight. FTO is highly abundant in adipose tissue and targets multiple genes to regulate adipogenesis. For instance, FTO directly targets Atg5 and Atg7 and mediates their expression in an m^6^A-dependent manner during adipogenesis. These two genes are also targets of YTHDF2 73. In porcine intramuscular pre-adipocytes, FTO can promote adipogenesis by inhibiting the Wnt/β-catenin signaling pathway 74. The effect of FTO on adipogenesis also appears to be regulated by enhanced expression of the pro-adipogenic short isoform of runt-related transcription factor 1 (RUNX1), which can promote adipocyte proliferation 75. FTO can affect the binding of alternative splicing regulator SRSF2 to m^6^A by regulating the m^6^A methylation level, thus mediating the alternative splicing of key genes involved in adipogenesis and regulating the differentiation of preadipocytes 76. In addition, FTO regulates adipogenesis by controlling cell cycle progression in an m^6^A-YTHDF2-dependent manner 77. The transcription factor zinc finger protein (Zfp217) modulates m^6^A mRNA methylation by activating FTO and CCND1 expression to promote adipocyte differentiation in an m^6^A-dependent manner 78, 79. In contrast, WTAP, METTL3, and METTL14 are negatively related to adipogenesis by promoting cell cycle transition in mitotic clonal expansion 80. METTL3 expression increases significantly in interscapular brown adipose tissue (iBAT) after birth and is an essential regulator of iBAT postnatal development and energy homeostasis 81. In porcine bone marrow stem cells, METTL3 represses adipogenesis via an m^6^A-dependent pattern 82. These results provide insight into the critical roles of m^6^A methylation during adipogenesis and suggest that m^6^A may be a novel potential biomarker of obesity.

Taken together, these findings demonstrate pivotal roles of the m^6^A modification in development. In the remainder of this review, we focus on the current understanding of the roles of m^6^A in skeletal muscle development and discuss potential future research directions.

## The regulation of skeletal muscle development

**Figure 2:**
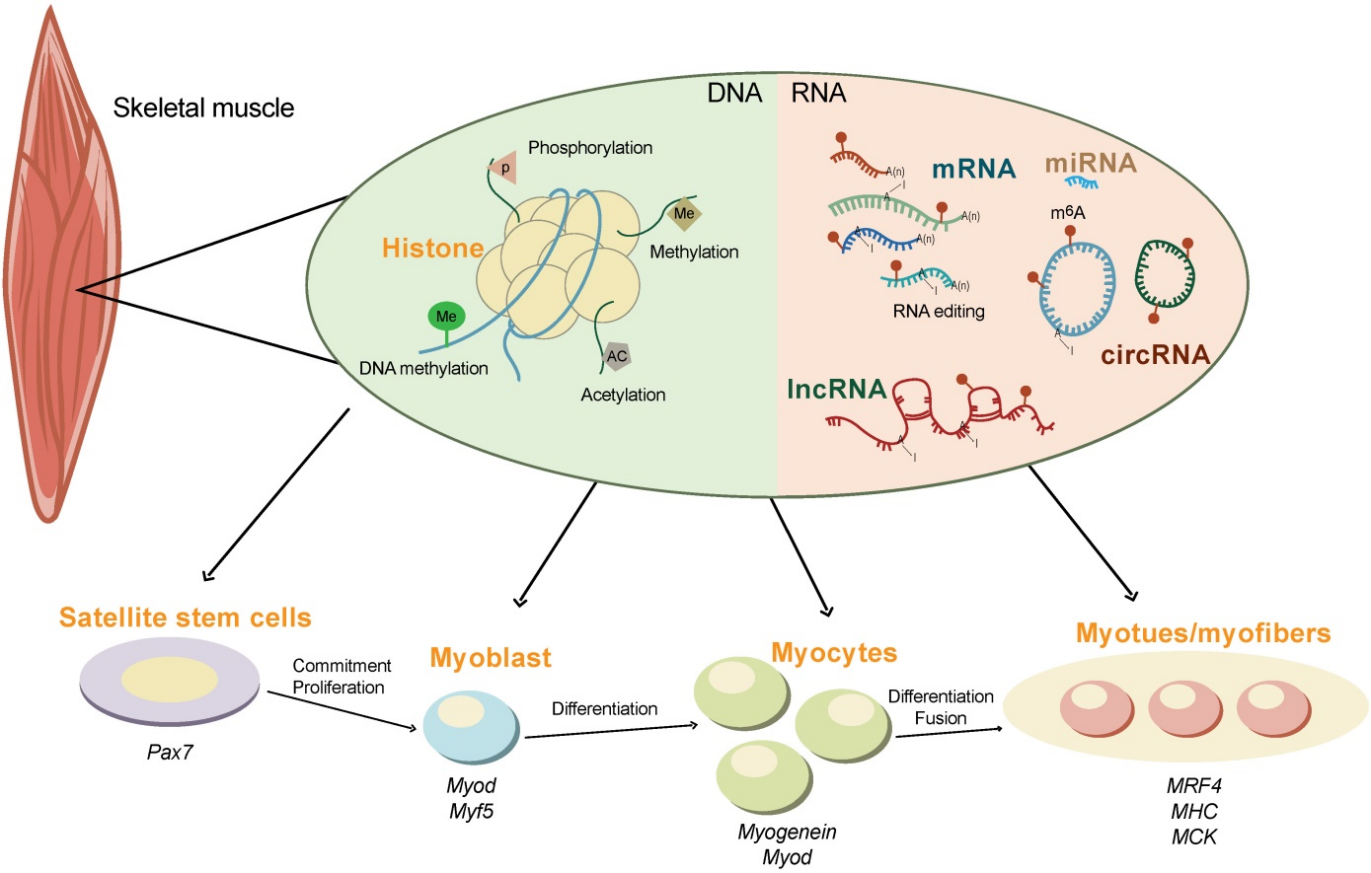
** The epigenetics regulation of skeletal muscle development.** The development of skeletal muscle is tightly controlled by multiple epigenetic modifications at both the DNA (e.g., histone modification and DNA methylation) and the RNA (e.g., m^6^A methylation, non-coding RNAs, and RNA editing) levels. The interplay between m^6^A and other epigenetic modifications orchestrate the expression of myogenic genes during commitment, proliferation, differentiation, and fusion of skeletal muscle.

Skeletal muscle is the most abundant tissue, accounting for 30-40% of body mass in adult humans 83 and 35-60% of that in domesticated animals 84. In animal husbandry, the development of skeletal muscle is directly associated with meat production, which is one of the most important economical traits 85. It is well known that skeletal muscle development is an extremely complex regulatory process orchestrated by myogenic genes and transcription factors (Figure 2). These transcription factors precisely control the proliferation, differentiation, and fusion of muscle cells according to strict time-sequence expression 86-88. The myogenic regulatory factor (*MRF*) family plays a decisive regulatory role in the fate of myoblasts and muscle development, constituting a family of basic helix-loop-helix transcription factors. The four members of this family are myogenic differentiation 1 (MyoD), myogenic factor 5 (MyF5), myogenic protein (Myogenin), and MRF4 89. MyF5 and MyoD are muscle-derived determinants of myoblast regulation and differentiation, while Myogenin and MRF4 are muscle-derived differentiation factors that induce terminal differentiation and play an important role in the development of skeletal muscle in the early embryo 90. Myogenin is the downstream gene of MyF5 and MyoD and is necessary for the fusion of mononuclear myoblasts to form multinucleated myotubes 91. MRF4 plays an important role in the terminal differentiation of muscle cells and in the maintenance of muscle fibers 92.

In addition, non-coding RNAs (ncRNAs) also play important regulatory roles in skeletal muscle development (Figure 2). The miRNAs, miR-1, -133, and -206, are specifically expressed in skeletal muscle and myocardium (myomiR). miR-1 and miR-206 promote skeletal muscle cell differentiation by inhibiting the expression of *Pax7* 93. miRNA-133 inhibits the differentiation of myoblasts and promotes myoblast proliferation by targeting the serum response factor (SRF) gene 94. Other miRNA, such as miR-148a and miR-21 are also reported to regulate skeletal muscle development 95, 96.

LncRNAs account for the vast majority of transcripts in the mammalian genome. LncRNA-H19 was the first lncRNA to be identified in mammals. The first exon of lncRNA-H19 can transcribe miR-675-3p and miR-675-5. These two miRNAs can downregulate the expression of Smad family members 1 and 5 (SMAD1 and SMAD5) and cell division cycle 6 (CDC6), inhibiting the proliferation and promoting the differentiation of C2C12 myoblasts 97, 98 Linc-MD1, the first identified muscle-specific lncRNA, is required for myoblast differentiation by regulating the levels of myocyte enhancer factor 2C (MEF2C) and mastermind-like protein 1 (MAML1) via the sponging of endogenous miR-133 and miR-135 in the cytoplasm 99.

CircRNAs exist widely in eukaryotes and lacks a 5'-terminal cap and a 3'-terminal poly (A) tail, forming a circular structure with covalent bonds 100. Studies have found that circRNAs are mainly involved in the regulation of muscle development by acting as molecular sponges of miRNA and encoding small peptides 101. For example, myocyte-specific circRNA circZNF609 encodes small peptides that participate in muscle differentiation and myoblast proliferation. Downregulation of circZNF609 expression during myoblast differentiation can promote myoblast proliferation 102.

These information provide a comprehensive profile for understanding the mechanisms underlying skeletal muscle development, and highlight the important functions of ncRNAs in skeletal muscle development. ncRNAs have a great potential to become therapeutic targets for muscular diseases and molecular markers for animal breeding. However, in comparison with the above regulators, the precise roles of m^6^A modification in the skeletal muscle and its temporal control during skeletal muscle development are still limited.

## The function and mechanism of m^6^A during myogenesis *in vitro*

**Figure 3:**
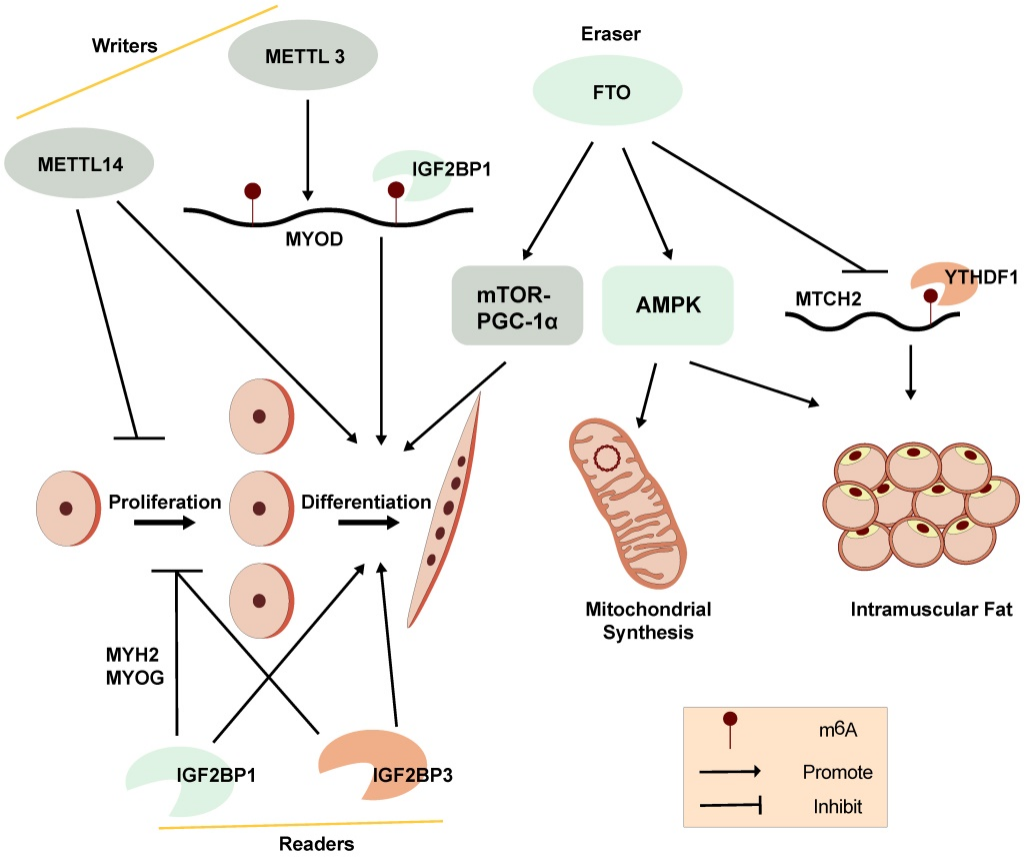
A diagram showing the functions and regulation of m^6^A and its regulators in myogenesis and skeletal muscle development.

Using mouse C2C12 myoblasts and muscle-specific adult stem cells (MuSCs) as models, recent studies have suggested that RNA m^6^A methylation plays an important role in myoblast proliferation and differentiation *in vitro* (Figure 3). The m^6^A modifications are closely linked to genes associated with transcriptional regulation during proliferation, and positively regulate myogenic differentiation in C12C12 skeletal myoblasts 103, 104.

A distinct m^6^A modification profile between proliferating and differentiating C2C12 myoblasts is observed. The global m^6^A levels decrease when C2C12 myoblasts transition from proliferation to differentiation. METTL3 regulates m^6^A levels in MuSCs and myoblasts, controlling their transition to different cell states. Knockdown of METTL3 can slow the proliferation of primary MuSCs in mouse and enhance their engraftment 103. Meanwhile, m^6^A modifications have been found to be enriched within the 5'-UTR of MyoD mRNA. METTL3 can stabilize MyoD mRNA by promoting mRNA processing in proliferative myoblasts for skeletal muscle differentiation. Knockdown of METTL3 can specifically downregulate the expression of processed MyoD mRNA in skeletal myoblast (Figure 3) 105. In addition, knockdown of METTL14 inhibits the differentiation and promotes the proliferation of C2C12 myoblasts 14.

Demethylase FTO is not only associated with obesity but can also regulate mTOR-PGC-1α-mediated mitochondrial synthesis and promote muscle cell differentiation through its m^6^A demethylase activity (Figure 3). The expression of FTO increases during myoblast differentiation, while silencing of FTO inhibits differentiation. Depletion of FTO leads to impaired differentiation and fusion* in vitro* 106. Meanwhile, overexpression of FTO could suppress the expression of mitochondrial carrier homology 2 (MTCH2), which is a target of YTHDF1. m^6^A enhances MTCH2 protein expression *via* an YTHDF1-dependent pathway and promotes adipogenesis in porcine muscles 107. The energy sensor AMP-activated protein kinase (AMPK) gene is a key regulator of skeletal muscle lipid metabolism. The level of mRNA m^6^A methylation in skeletal muscle is negatively correlated with skeletal muscle lipid content. AMPK regulates skeletal muscle lipid accumulation through fat quality and obesity-related protein by FTO-dependent m^6^A demethylation 108. Our recent studies suggest that the m^6^A methylation readers, IGF2BP1 and IGF2BP3, both promote myoblast differentiation and repress proliferation in C2C12 myoblast cells 14, 109. These findings provide further understanding of the potential functions of RNA m^6^A methylation on myogenesis.

However, it's confused that the m^6^A regulators, including m^6^A methyltransferase (METTL3 and METTL14), demethylase FTO and readers (IGF2BP1 and IGF2BP3), are reported to promote myoblast differentiation in C2C12 myoblasts (Figure 3) 14, 106, 109, indicating that the epigenetic regulation of m^6^A modifications in myogenesis is a complex regulatory networks and might be affected by other genes and regulatory elements at multi-levels. Due to the contradictory regulation of m^6^A modification on myogenesis, the correlation between m^6^A modification and myogenesis is still needed to be further explored. The function of other m^6^A regulators in myogenesis should also be studied.

## The dynamic change and potential roles of m^6^A methylation in skeletal muscle development

To date, a few studies have evaluated the genome-wide m^6^A modification on skeletal muscle tissue *in vivo*, the dynamic change of m^6^A methylation in skeletal muscle development and the correlation between m^6^A and skeletal muscle are explored 14, 103, 110. Many muscle-specific m^6^A sites have been identified in mammals 41, 110, 111. In mouse, it's reported that the maternal high-fat diet alters the m^6^A pattern in skeletal muscle and adipose in a development-dependent way 112. Using liquid chromatography/mass spectrometry (LC-MS) and MeRIP-seq analysis, they find that global m^6^A levels increase during the early stages of skeletal muscle regeneration. In the *longissimus dorsi* muscle of Landrace pigs (lean-type breed), the global m^6^A level and the expression of METTL3 at both mRNA and protein levels are much higher than those of Jinhua pigs (obese-type breed), while the level of FTO shows the opposite trend, suggesting that the m^6^A level is negatively associated with fat deposition in skeletal muscle 107. Depletion of FTO impairs postnatal skeletal muscle development *in vivo* 106. The MeRIP-seq analysis of skeletal muscle in wild boar, Landrace and Rongchang (obese-type breed) pigs suggests that m^6^A modification occurs in a breed-differential pattern 110.

Moreover, using a refined MeRIP-seq method that is optimal for use with samples containing small amounts of RNA, our group recently performed transcriptome-wide m^6^A profiling at six prenatal developmental stages in porcine skeletal muscle, ranging from 33 to 90 days *post coitus* (*dpc*) 14. A highly dynamic change in genome-wide m^6^A modification is observed across different developmental stages. RNA immunoprecipitation assay reveals that IGF2BP1 targets many myogenesis genes in skeletal muscle, such as MYH2 and MyoG, suggesting that IGF2BP1 regulates skeletal muscle development via binding to m^6^A-modified target genes 14. The results also show that most of the m^6^A modified genes are commonly methylated throughout prenatal skeletal muscle development, and the differentially methylated genes are enriched in pathways related to skeletal muscle development 14. Interestingly, we also find that DNA methylation regulates the expression and function of IGF2BP3 by modulating transcription factor SP1 binding in skeletal muscle 109.

Overall, these studies generally profiled the dynamic change of m^6^A modification in skeletal muscle and highlighted their correlation between m^6^A modification and skeletal muscle development. However, skeletal muscle is highly heterogeneous and consists of a variety of cells that have a network of crosstalk 113, 114, the function and regulation of m^6^A modification *in vivo* and *in vitro* might be biased*.* Single-cell analysis will be helpful to understand the roles of m^6^A modification in different skeletal muscle cell types in the future, thought this technology is currently not available in m^6^A. In addition, further* in vivo* studies in individuals using genetically modified mouses of m^6^A regulators as models are necessary to determine the function and mechanism of m^6^A in skeletal muscle development.

## Closing remarks and outlook

Here, we summary the function and mechanism of m^6^A modification, and highlight the current status of knowledge regarding the roles of m^6^A in myogenesis skeletal muscle development, which helps to further understand the mechanism underlying the epigenetic regulation of skeletal muscle development and muscle-related diseases. We suggest that the m^6^A modification can be used as a target for the therapy of muscle diseases and a biomarker in animal breeding for the improvement of meat production traits in the future.

However, in comparison with other tissues, such as the brain and adipose, knowledge regarding the biological function and regulatory mechanism of m^6^A in skeletal muscle remain limited. Several avenues of research should be explored in the future. Firstly, although several studies have revealed the dynamic RNA m^6^A profiles during skeletal muscle development, only a few developmental stages or samples were used. For example, the largest study assayed 18 samples at six prenatal developmental stages spanning 33-90 *dpc* in pigs 14. Future studies should elucidate the m^6^A methylome profiles of skeletal muscle spanning a wider range of developmental stages, including both prenatal and postnatal stages in mammals. Secondly, considering the difficulties in sampling skeletal muscle at the embryonic stages, future studies should develop novel methods that use small amounts of RNA to profile the genome-wide m^6^A maps at a higher resolution. Moreover, only bulk skeletal muscle can currently be used for genome-wide m^6^A methylation analysis. The fiber composition and non-muscle cells such as vasculature and immune cells may partly influence the measurement of m^6^A modifications. It is the hope that single-cell sequencing technology may be used to profile the m^6^A transcriptome map at the genome-wide level in the near future. Thirdly, recent studies performed in skeletal muscle have mainly focused on the function of the m^6^A modification in mRNA; however, the distribution and biological function of m^6^A in ncRNAs (such as lncRNAs and circRNAs) and regulatory elements (such as enhancers and promoters) remain unclear. Fourthly, adenine methylation is well known as a common mechanism for controlling gene expression. Knockout or overexpression of FTO and ALKBH5 only slightly changes the overall level of m^6^A, suggesting that there exist other unknown m^6^A demethylases that await discovery. The upstream regulators and small molecules of m^6^A enzymes are needed to be identified. Moreover, the regulatory mechanisms underlying the actions of m^6^A methyltransferases, demethylases, and binding proteins on m^6^A to spatiotemporally regulate downstream pathways and mediate skeletal muscle development remain largely unclear. Further investigation into targeted genes and m^6^A modifications using gene editing and epigenetic tools, such as CRISPR/cas9 technology, will be required to elucidate the mechanisms of dynamic m^6^A modification during skeletal muscle development. Finally, in addition to m^6^A, there exist many other epigenetic modifications in the genome and transcriptome; therefore, the interplay between m^6^A and other epigenetic modifications during skeletal muscle development should be explored. Integrative multi-omics will be a useful approach to comprehensively understand the regulatory mechanism of skeletal muscle development.
